# Bioaerosols, Noise, and Ultraviolet Radiation Exposures for Municipal Solid Waste Handlers

**DOI:** 10.1155/2017/3081638

**Published:** 2017-01-12

**Authors:** France Ncube, Esper Jacobeth Ncube, Kuku Voyi

**Affiliations:** School of Health Systems and Public Health (SHSPH), Faculty of Health Sciences, University of Pretoria, Pretoria, South Africa

## Abstract

Few studies have investigated the occupational hazards of municipal solid waste workers, particularly in developing countries. Resultantly these workers are currently exposed to unknown and unabated occupational hazards that may endanger their health. We determined municipal solid waste workers' work related hazards and associated adverse health endpoints. A multifaceted approach was utilised comprising bioaerosols sampling, occupational noise, thermal conditions measurement, and field based waste compositional analysis. Results from our current study showed highest exposure concentrations for Gram-negative bacteria (6.8 × 10^3^ cfu/m^3^) and fungi (12.8 × 10^3^ cfu/m^3^), in the truck cabins. Significant proportions of toxic, infectious, and surgical waste were observed. Conclusively, municipal solid waste workers are exposed to diverse work related risks requiring urgent sound interventions. A framework for assessing occupational risks of these workers must prioritize performance of exposure assessment with regard to the physical, biological, and chemical hazards of the job.

## 1. Introduction

Municipal solid waste management is a vital activity in the context of protecting human health and environment [1–3]. Municipal solid waste management workers perform various tasks such as street sweeping [4–6], manually loading waste into waste collection vehicles [7, 8] and driving such vehicles [9–11]. Such activities expose these workers to various occupational health risks associated with the characteristics of the waste they handle [12–14], the waste collection methods used [15–17], and the state of the working environment [8, 18–20].

Whilst over the past two decades valuable evidence has accumulated on the occupational dust and noise levels in the mining [21–23], manufacturing [24, 25], and agricultural sectors [26–28], very few studies have investigated such hazards for municipal solid waste workers. Of these few studies, the majority of them have been conducted in industrialized countries. The results of epidemiologic studies obtained in developed countries cannot be extrapolated with complete confidence to developing countries [29]. Our study, conducted in a developing country, envisages enriching the existing body of knowledge on hazards of municipal waste handlers.

Additionally, most of these few studies primarily focused on waste recycling plants [30–32], composting plants [33–35], and hazardous landfills [36] rather than waste workers responsible for loading municipal solid waste trucks, municipal landfilling operations, central waste collection systems, and street cleansing. Resultantly, very little has been documented on the occupational health risks of municipal solid waste workers. This suggests that such workers are currently exposed to more or less unknown dust, bioaerosols, and noise levels that may endanger their health. Notably a point of congruence among previous studies is the notion that to date little has been done to characterize biological hazards associated with waste collection [15, 37, 38].

It is dust which is in the breathing zone or entering the respiratory system which may pose health risks to the employee and should therefore be assessed and monitored [25]. The impetus to the determination of bioaerosols in the present study is the growing body of evidence in contemporary literature associating bioaerosols from organic dust with respiratory complaints on waste workers [12, 39, 40]. Furthermore, a key recommendation arising from Heida et al. [41] is the need to determine the organic dust concentrations from waste.

Additionally, assessments of workplace noise exposures are justified on the basis that noise hazards are globally ranked among the top five occupational stressors with grave repercussions on the worker and the organisation [42–44]. Notably, previous literature has strongly associated continuous occupational noise exposures of 85–90 dB (A) with adverse health conditions on the worker such as Noise Induced Hearing Loss (NIHL) [45, 46]. Liu et al. [47] further propound that the large machinery used to dig, transport, and compact landfills can generate noise level higher than 85 dB.

In both industrialized and developing countries, very little research is available on the thermal conditions in which waste workers work. More importantly, in tropical countries summer outdoor temperatures can be unbearably hot. Such high temperatures may render outdoor activities such as municipal solid waste collection, street sweeping, and landfilling operations a health hazard due to increased risk of excessive sweating, headaches, heat stress, offensive odours, and fly infestation from decomposition processes of organic waste fractions. Conversely, cold outdoor temperatures have been associated with frost bite [48] and hypothermia among gardeners [49].

Consequently this paper aims to determine the occupational dust, noise, and thermal exposures in the field of municipal solid waste management so as to build up the much needed evidence for developing a generic framework for assessing occupational health risks of municipal solid waste handlers.

## 2. Materials and Methods

Personal sampling was performed using two field monitors mounted in the breathing zone of workers, approximately 1.5 m above ground level. One monitor was for collecting of total dust and the other for bioaerosols (bacteria and fungi). Environmental samples were collected using similar equipment mounted at the breathing zone, from the active landfilling sites, truck cabins, and street cleaning sites. Total dust and bioaerosols samples were collected from various sites (Table 1). Culturing for Gram-negative bacteria (GNB) was done at 37°C for 24 hours using the McConkey media whist for viable fungi the Malt Extract Agar media diluted with 0.01% chloramphenicol was used. The occupational noise exposure doses were measured using the Quest sound level meter model SOUNDPRO SP-DL-1/3n and the ultraviolet thermal conditions were determined using the AZ thermoanemometer instrument. Qualitative data on health complaints of waste workers was gathered using self-administered questionnaires. Key informant interviews with waste managers in local government structures, in the Health Department and the Environmental Management Agency, were conducted to tap on the richness and diversity of expert input on waste management health risks. Collected data was analyzed using STATA version 13.

## 3. Results and Discussion

### 3.1. Total Dust and Bioaerosols Exposures

Our study found high mean exposure concentrations for total dust, Gram-negative bacteria (GNB), and fungi for personal samples collected from refuse bin loaders and truck cabin samples (Table 1). This suggests the priority for exposure assessment with regard to total dust and bioaerosols should be focused on waste loaders and the truck cabins. Regular proper cleaning, drying, and aeration of the waste collection vehicle are essential so as to safeguard the respiratory health of waste workers.

**Figure 1:**
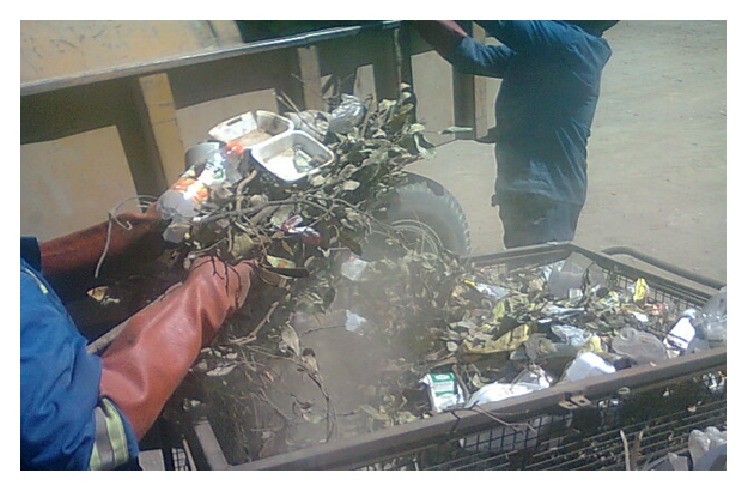
Dust generation from loading mixed waste without proper containment bags.

The high mean total dust, Gram-negative bacteria (GNB), and fungi exposure concentrations reported in our study for waste loaders and truck cabin may be attributed to the processes of manual offloading and loading of mixed waste streams without proper containment bags (Figure 1).

**Figure 2:**
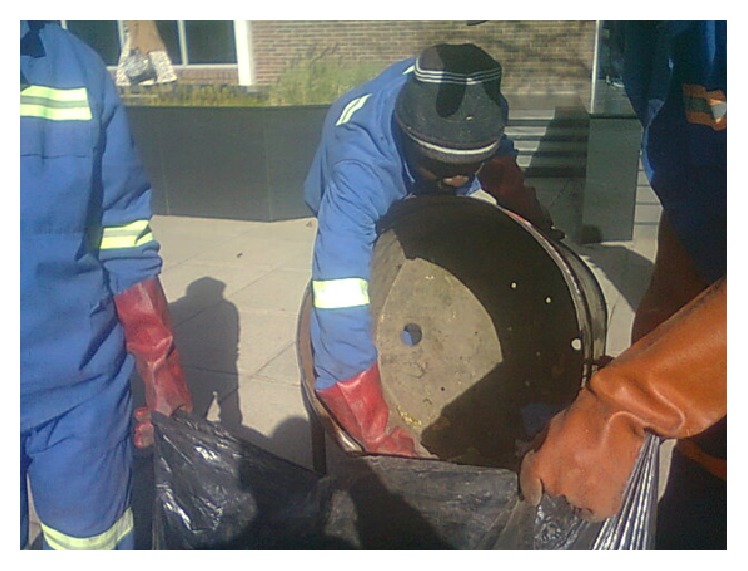
Process of transferring of waste from stationery bin to plastic bags.

Our study argues that transferring of waste from stationery bins to other bags (Figure 2) results in almost double exposures to dust and bioaerosols for each refuse collector: firstly during transferring process from one bin to the other and secondly during the bin empting process into the truck.

Nonstationary bins that are directly emptied into the refuse collection truck could avert exposures encountered during the waste bin transferring process. Alternatively, all stationery bin containers may be fitted with removable bin liners that are directly emptied into the waste collection trucks.

Our study found high mean exposure concentrations for total dust, Gram-negative bacteria (GNB), and fungi for personal samples collected from refuse bin loaders and for truck cabin samples (Table 1). Also for all the sampled waste sites concentrations of both Gram-negative bacteria (GNB) and fungi were basically in the order of 10^3^. Similarly the available body of research on bioaerosols from municipal solid waste management activities has reported the mean and range of bacterial and fungal cells per m^3^ of air between the orders of 10^3^and 10^6^. Recently, in Copenhagen, Madsen et al. [50] found bacterial cells per cubic meter of air to be in the range of 112 to 4.8 × 10^4^ for waste collector's personal samples and 48 to 2.6 × 10^3^ for truck cab samples.

Park et al. [11] reported exposure levels among Korean waste collectors ranging between 0.13 × 10^5^ and 23.5 × 10^5^ for total viable bacteria and 2.4 × 10^4^ CFU/m^3^ and 10.8 × 10^4^ CFU/m^3^for fungi. In Norway, Heldal et al. [51] observed slightly higher summer bacterial and fungal exposures for waste collectors (0.4 × 10^6^ cells/m^3^ to 3.6 × 10^6^ cells/m^3^) compared to winter exposures (0.4 × 10^6^ cells/m^3^ to 2.0 × 10^6^ cells/m^3^). Lavoie and Dunkerley [37] reported mean bacterial concentrations of 10^3^ to 10^4^ CFU/m^3^ and mean fungal concentrations ranging between 8.3 × 10^3^ CFU/m^3^and 9.8 × 10^4^ CFU/m^3^, among waste collectors in Canada. In a sample of German waste collectors, Neumann et al. [52] reported bacterial concentrations ranging between insignificant levels and 10^6^ CFU/m^3^ and fungi concentrations between 8.3 × 10^3^ CFU/m^3^ and 9.8 × 10^4^ CFU/m^3^. Evidently, municipal solid waste handling processes expose waste workers to bioaerosols from organic dust and such exposure may precipitate onset of respiratory problems [9, 12, 53].

### 3.2. Noise Exposures

The highest average noise levels (84.86 dBA) were recorded in the central waste collection points whilst the lowest (83 dBA) was in the cabin of waste collection truck. In all measured waste management sites (Table 2) mean noise levels were within the international threshold limit value (85 dBA).

However, the major sources of noise were waste collection vehicles' running engines, other traffic and landfilling vehicles. Constituents of municipal solid waste such as glass and metal tins also contributed to the occupational noise particularly during emptying of metal bins on the metal floor of waste collection vehicles. High working speed with regard to offloading of waste bins tended to produce a monotonous noise. Additionally, Jerie [54] observes that, for informal waste workers, sources of noise entail working closer to heavily frequented roads and other noise sources such as carpentry, metal work, and engineering workshops. Unfortunately, all the refuse collection vehicles in the present study had no noise reduction mechanisms such as rubber lined floors.

Our study findings are far below personal noise levels observed in glass waste collection operations (108 to 131 dB L_AE._) in the United Kingdom [55]. According to Kuijer and Frings-Dresen [17], Stassen and colleagues found personal noise exposure levels as high as 96.4 dBA among waste collectors in the Netherlands. Evidently the noise risk exposure differs per each scenario which suggests the need for each authority responsible for waste management to consider performing its own noise assessments so as to yield relevant data for informing decision making on required interventions.

In the present study we observed that none of the municipal solid waste workers wore any hearing protection devices though two workers in the waste collection crew complained of occasional temporary hearing loss. Whilst our study did not find mean noise levels above recommended levels we suggest that where hearing protection devices are considered as precautionary measures, there is need to consider the possibility of failure to hear warning sounds from other road users which may increase the risk of accidents and injury among waste workers.

### 3.3. Thermal Conditions

Outdoor work is associated with greater exposure to hot and cold temperatures [49]. In our present study we observed that municipal solid management operations such as street sweeping, landfilling activities, and door to door waste collection are performed in such hot environments, often in unshaded areas. We observed mean summer temperatures higher than 33°C in most waste management areas (Table 3).

Temperatures below and above those typically preferred by most people have a significantly detrimental effect on the safety related behavior of workers [56]. This suggests that for optimum promotion of occupational safety behavior of municipal solid waste workers the thermal working environment requires attention by responsible authorities. In our present study we observed that most waste workers complained of headaches, sunburn, heat stress, excessive sweating, dehydration, and difficulties in concentration in assigned tasks. Notably difficulties in concentration may increase the risk of being run over not only by waste collection vehicles but also by other traffic especially during the day when the traffic volume is high. Inhalation of toxic emissions like carbon monoxide and carbon diode from traffic exhausts pipes may further exacerbate the situation. This suggests the need for waste managers, particularly in tropical countries, to consider rescheduling summer waste collection services for early morning hours or at night when temperatures are cooler and low and traffic volume low. Additionally, waste workers need to be encouraged to take regular breaks and rest in cooler shades where oral rehydration fluids can be given to refresh them.

### 3.4. Other Hazards

The current study found that on average the monthly total amount waste collected in the study area, in tones, was on average 566.08 of which 518.88 was from residential suburbs, 18.88 was from commercial enterprises, and 28.32 was from industries. Results from the physical waste compositional analysis revealed that residential waste on average constituted 24% food waste, metal containers 4%, glass and ceramics 2%, diapers 2%, toxic waste streams 1%, plastics and paper 13%, and 54% miscellaneous waste streams. The commercial waste stream was mainly dominated by food waste 42%, metal tins containers 24%, glass 1%, paper and plastics 7%, and other waste streams 24%. Evidently wastes from both the residential and commercial sources had significant proportions of biodegradable food waste. Biodegradation of such organic waste produces offensive odours and supports fly breeding and infestation particularly in summer when temperature is high. The presence of diapers, though in small proportions, in residential waste is a cause of concern since this poses risks of transmission of pathogenic organisms into waste workers' hands. Waste from the industries was mainly scrap metals, rumble, glass, and food remains.

### 3.5. Hazardous Waste Streams

Results from the present study revealed that the major toxic waste streams in municipal solid waste included hair sprays, shampoos, expired medicines, pesticides and e-waste, shoe and floor polish, carpet and furniture cleaning agents, motor vehicle brake fluid, battery acid, and nail paints. Although available in small quantities (1%), toxic waste inevitably renders the entire municipal solid waste potentially toxic and can lead to various occupational health risks for waste collectors through inhalation, ingestion, and dermal exposure pathways. Pesticide residues such as organophosphates could affect the central nervous system through inhibition of the choline esterase enzyme. In the present study we observed discarded pesticide containers in household waste streams which could be a source of arsenic exposures for waste workers. The International Agency on Research on Cancer [57] categorises arsenic as a known human carcinogen.

Arsenic can lead to cellular toxicity [58–60], neurotoxicity [56, 61], immunotoxicity [62], cardiovascular diseases [57], and developmental and reproductive toxicity [61, 63, 64]. Additionally, our study reports e-waste streams in household municipal solid waste streams.

The major e-waste components found in the present study include fluorescent and nonfluorescent bulbs, circuit boards, lead and acid car batteries, printer inks and tonner, spark plugs, motherboards, keyboards, monitors, electrical switches, and thermostats. Fluorescent and nonfluorescent bulbs, circuit boards, and car batteries in municipal solid can be source of lead (Pb) and mercury (Mg). Similarly, inks and tonner for printers and NiCd rechargeable batteries can be a source of cadmium (Cd). Also, monitors and keyboards in municipal solid waste streams are a cause of concern since they can be primary sources of polyvinyl chlorides (PVC) which may emit harmful gaseous substances such as hydrogen chloride gas.

Previous literature has associated lead (Pb), cadmium (Cd), mercury (Mg), and polyvinyl chlorides from e-waste with various adverse mental health effects such as cognitive disturbances and reduced intelligence quotient (IQ) [65–69]. Most of these chemicals from e-waste have been found to heavily contaminate ambient air [68, 70, 71]. Such high ambient air concentrations of toxic e-waste chemicals could lead to relatively high inhalational exposures for waste workers. This suggests the need for waste managers to periodically conduct exposure monitoring for waste workers and engage the generators of hazardous waste in efforts such as extended producer responsibility in sound waste management, Life Cycle Analysis (LCA), and regulatory compliance.  Table 4 summarises the observed hazardous waste streams and potential occupational risks for municipal solid waste workers for the current study.

Results from the physical waste compositional analysis revealed considerable proportions of infectious materials in municipal solid waste (2%), such as diapers and used tissue (Table 4). Contact with such contaminated materials may contribute to transmission of faecal-oral diseases such as hepatitis A. Previous work has richly detailed the hepatitis A (HAV) risk associated with waste management. In Greece, Dounias and Rachiotis [72] observed a significantly increased prevalence of HAV infection among solid waste collectors and suggested among other measures vaccination of waste workers against HAV.

**Figure 3:**
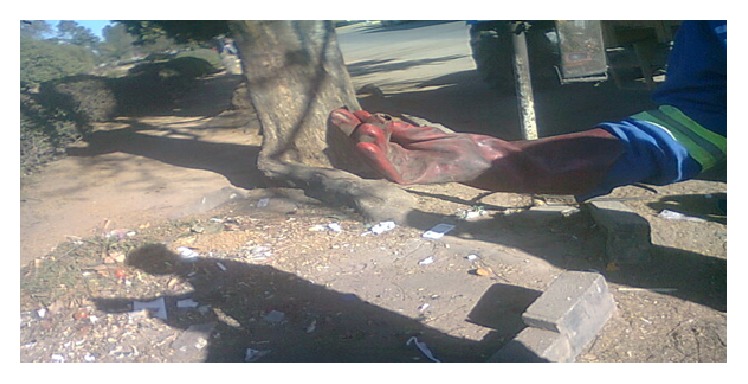
Waste collector wearing nonpuncture-proof and worn out gloves.

Our study found relatively high levels of mechanical waste components such as scrap metal, broken glass, and razor blades (Table 4). Such components have potential to inflict physical harm to municipal solid waste workers in form of cuts, open wounds, and bruises. Although gloves were provided for waste workers, they were not puncture-proof and were worn out (Figure 3). In light of epidemiologic evidence linking hepatitis B and needle stick injury [73], such an unhealthy status of waste workers' gloves is a cause of concern. Tsovili et al. [73] found significantly higher prevalence of hepatitis B virus infection (*p* < 0.01) in waste collectors (15%) in comparison with the control group (2.5%). Thus municipal solid waste collection is a job heavily laden with biological risks that may endanger waste workers' health.

### 3.6. Strengths and Limitations

Our paper presents the strength of the inclusion of occupational hygiene measurements related to several occupational hazards. Particularly, bioaerosols exposure determination, occupational noise, and thermal conditions measurement were done. However we did not measure exposures through other routes such as hand contact with contaminated materials. Thus our study is unable to estimate the microbiological risk through the ingestion route since we did not swab waste workers' hands and nails to determine the remaining concentrations of* E. coli* and faecal after washing their hands. Nonetheless since results from our physical waste compositional analysis revealed faecal waste (diapers) in municipal solid waste streams (Table 4), waste workers may be at risk of faecal-oral transmitted diseases through contaminated hands following handling of such waste materials. Therefore, further studies may need to determine the efficiency of hand washing methods utilised by municipal solid waste workers.

To the best of our knowledge, very limited studies have been conducted on the GNB and fungi exposure concentrations at municipal solid waste management sites such as active landfilling sites, refuse bin collection points, and truck cabins. Our study enriches and broadens the existing body of knowledge in this negated area (Table 4). Moreover, our findings have positive implications on the planning and conduction of municipal solid waste collection activities. Some proposed changes include rescheduling summer waste collection for early morning or at night when temperatures are cooler, provision of resting shades, and oral rehydration fluids to cushion waste workers from heat exhaustion and syncope.

Other shortcomings of our current study entail usage of relatively small samples sizes (Table 1) and a cross-sectional study which may limit our findings with regard to proving causality relationships and extrapolating our results to the wider population of municipal solid waste workers.

## 4. Conclusion

Our study found high mean exposure concentrations for total dust, Gram-negative bacteria (GNB), and fungi for personal samples collected from refuse bin loaders and for truck cabin samples. This suggests the priority for exposure assessment with regard to total dust and bioaerosols should be focused on waste loaders and the truck cabins. Also, we observed mean summer temperatures higher than 33°C in most waste management areas and workers complained of headaches, sunburn, heat stress, excessive sweating, dehydration, and difficulties in concentration in assigned tasks. Consequently our study argues that in tropical countries it is better to perform summer waste collection services in early morning hours or at night when temperatures are cooler. Waste workers should be encouraged to take regular breaks and rest in cooler shades where oral rehydration fluids can be given to refresh them. In light of the results from our physical waste compositional analysis, our study concludes that municipal solid waste workers are exposed to diverse toxic, mechanical, and infectious hazards requiring sound mitigation measures.

## Figures and Tables

**Table 1:** Total dust and bioaerosols exposures (mean and range) in different working areas.

Sampling site	Description	(*n*)	Total dust mg/m^3^	GNB 10^3^ cfu/m^3^	Fungi 10^3^ cfu/m^3^
Site A	Bin loaders	12	8.2 (0.8–26)	1.5 (0.16–6.8)	66 (7.2–136)
	Drivers	4	4.2 (0.8–12)	1.6 (0.2–2.8)	36 (6.4–68)
	Skip bins	4	3.2 (0.6–10)	1.2 (0.1–6.4)	28 (5.8–62)

Site B	Truck cabin samples	4	8.6 (0.9–26)	1.6 (0.18–6.8)	68 (6.4–12.8)

Site C	Site workers	12	0.4 (0.2–0.8)	6.8 (0.04–28)	3.2 (0.4–8.2)
	Machine operators	4	0.6 (1.4–2.2)	22 (0.6–120)	21 (0.3–100)
	Site samples	4	0.3 (0.1–0.8)	6.2 (0.02–24)	2.8 (0.2–7.4)

Site D	Sweepers	12	0.08 (0.04–0.3)	ND	12 (14–24)
	Site samples	4	0.04 (0.02–0.5)	ND	8 (12–22)

ND: none detected; GNB: Gram-negative bacteria; cfu: colony forming units; A: waste collectors; B: truck cabin; C: active landfilling site; D: street cleaning; *n*: sample size.

**Table 2:** Noise level measurements (dBA) in various working areas.

Site	Site description	Average noise value (dBA)	ISO standard (dBA)
A	Noise mainly generated by hydraulic waste collection trucks and passing traffic.	84.86	85
B	Waste spreading in cells and soil cover application. Noise generated by waste compactors.	84.32	85
C	Manual offloading of waste bins into waste collection vehicles. Noise mainly generated by hydraulic waste collection trucks and passing traffic.	83.00	85

A: central collection points, B: active landfilling area, and C: offloading area into truck.

**Table 3:** Summer thermal conditions measured in various waste sites.

Work site	Average *T*^ °^C	Waste workers' concerns
Loading waste collection vehicles	33.34	Sweating, dehydration, heat syncope, and heat exhaustion.

Street and open areas sweeping	33.28	Loss of concentration. High risk of being run over by traffic, sweating, dehydration, and headache.

Manning waste disposal sites	33.29	Offensive odours and high fly infestation from increased organic, sweating, heat stress, and headaches.

Driving waste collection vehicles	26.25	Sweating and occasional headache.

**Table 4:** Household hazardous waste compositional analysis and associated hazards.

Waste type	Components	Potential hazards for waste handlers
Toxic (1%)	Hair sprays, lotions, shampoos, expired medicines, and pesticides	This can lead to systemic intoxication from inhalational exposures. This can also lead to severe burns from accidental or spontaneous ignition of flammable materials.
	E-waste (e.g., fluorescent bulbs, car batteries, printer ink, and tonner)	Toxic metals in e-waste may damage target organs leading to various toxicity effects.

Infectious (2%)	Diapers and used tissue	Infectious waste can transmit bacteria responsible for spreading diarrhoeal diseases. Biodegradation of faecal matter in diapers generates offensive odours that can induce anorexia, nausea, and vomiting.

Mechanical hazards	Scrap metal, broken glass, razor blades, and needles	This can cause injuries through piecing and bruises and facilitate transmission of hepatitis B.

Average % by weight calculated per weekly waste generation rates.
